# 3D Silk Fiber Construct Embedded Dual-Layer PEG Hydrogel for Articular Cartilage Repair – *In vitro* Assessment

**DOI:** 10.3389/fbioe.2021.653509

**Published:** 2021-03-24

**Authors:** Jung Soo Kim, Jaeho Choi, Chang Seok Ki, Ki Hoon Lee

**Affiliations:** ^1^Department of Agriculture, Forestry and Bioresources, Seoul National University, Seoul, South Korea; ^2^Research Institute of Agriculture and Life Sciences, Seoul National University, Seoul, South Korea

**Keywords:** silk fiber, fiber-reinforced hydrogel, 3D fiber construct, PEG hydrogel, dual-layer hydrogel, cartilage repair, chondrogenic differentiation

## Abstract

Since articular cartilage does not regenerate itself, researches are underway to heal damaged articular cartilage by applying biomaterials such as a hydrogel. In this study, we have constructed a dual-layer composite hydrogel mimicking the layered structure of articular cartilage. The top layer consists of a high-density PEG hydrogel prepared with 8-arm PEG and PEG diacrylate using thiol-norbornene photo-click chemistry. The compressive modulus of the top layer was 700.1 kPa. The bottom layer consists of a low-density PEG hydrogel reinforced with a 3D silk fiber construct. The low-density PEG hydrogel was prepared with 4-arm PEG using the same cross-linking chemistry, and the compressive modulus was 13.2 kPa. Silk fiber was chosen based on the strong interfacial bonding with the low-density PEG hydrogel. The 3D silk fiber construct was fabricated by moving the silk fiber around the piles using a pile frame, and the compressive modulus of the 3D silk fiber construct was 567 kPa. The two layers were joined through a covalent bond which endowed sufficient stability against repeated torsions. The final 3D silk fiber construct embedded dual-layer PEG hydrogel had a compressive modulus of 744 kPa. Chondrogenic markers confirmed the chondrogenic differentiation of human mesenchymal stem cells encapsulated in the bottom layer.

## Introduction

Articular cartilage is a highly hydrated tissue without nerves, blood and lymph vessels, lacking a general healing process. As such, any lesion in the articular cartilage requires a surgical operation. Currently, micro-fracture and autologous chondrocyte transplantation are operated on according to the degree of cartilage damage (Makris et al., 2015). However, such processes can only repair small areas. Therefore, researchers attempt to find proper biomaterials for articular cartilage repair (Grande et al., 2013; Karuppal, 2017).

Hydrogels have been recognized as suitable candidates for articular cartilage tissue engineering because they are similar to natural ECM in terms of their high water content (Vega et al., 2017). However, many hydrogels do not match the mechanical properties required for articular cartilage replacement. The most straightforward strategy to improve the mechanical properties of hydrogels is to increase the cross-linking density through physical or chemical cross-linking (Sun et al., 2012; Li et al., 2018; Zhang et al., 2018). In addition to solving the problem of mechanical mismatch between the hydrogel and articular cartilage, researchers also tried to mimic the multi-layered structures of articular cartilage because these structural elements also provide an important cellular environment for successful tissue reconstruction (Rose and De Laporte, 2018). In this respect, multi-layer hydrogels with different mechanical properties are proposed as candidate biomaterials for articular cartilage repair (Armiento et al., 2018; Liu et al., 2018).

Among the various polymers, poly(ethylene glycol)(PEG) is one of the most utilized polymers for hydrogels (Spicer, 2020). PEG is an attractive polymer due to the chemical and biological inertness, as well as its diversity in molecular weight (MW), topology (linear, branched, star-shaped), and chemical reactivity (Zhu and Marchant, 2011). The diversity of PEG in its chemical structure makes it possible to prepare PEG hydrogels with tunable mechanical properties and degradability (Li et al., 2018). For example, the cross-linking density, which significantly affects the mechanical properties, can be controlled using PEG chemistries such as 4-arms vs. 8-arms (Kim et al., 2016) and short- vs. long-chain (Borges et al., 2020). Therefore, we chose PEG for the manufacture of a dual-layer hydrogel having different cross-linking densities. Using the same polymer for both layers minimizes interfacial bonding problems when two different polymers are used for each layer. It has been reported that bi- or multi-layer PEG hydrogel forms a stable interfacial zone preventing delamination of different layers (Kinneberg et al., 2015; Aziz et al., 2017). In this study, a high-density PEG (HD-PEG) hydrogel will form the upper layer of the dual-layer hydrogel, acting like a protective layer against mechanical stress. The bottom layer of the dual-layer hydrogel will be made by a low-density PEG (LD-PEG) hydrogel with multi-capabilities such as biodegradability, stem cell encapsulation, and differentiation of stem cells into chondrocytes.

However, the LD-PEG hydrogel should be reinforced to dissipate the mechanical stress from the above layer. Further increasing the cross-linking density should be avoided since the fate of cells could be affected significantly (Malda et al., 2013; Simona et al., 2015; Hadden et al., 2017). A method to increase the stiffness of hydrogel without changing the cross-linking density of the polymer network is introducing reinforcement fillers such as fibers (Zhang et al., 2019; Beckett et al., 2020). Fibers prepared by electrospinning were frequently applied to reinforce the hydrogel. However, difficulties in preparing a load-bearing 3D construct would be the limitation of the electrospun fibers (Ki et al., 2007). A 3D construct of fibers could be prepared by 3D printing, such as fused deposition modeling (FDM). They could serve as a load-bearing layer, but the choice of polymer is limited since the melt viscosity should be low enough to allow printing but high enough to provide structural support (Wang et al., 2017). Here, we suggest a new type of 3D fiber construct made of readily available fibers. In FDM, thermoplastic filaments are melted and extruded as a fiber, which quite resembles the process of conventional melt spinning. However, besides material selection limitations, the process parameters of FDM are much more limited than melt spinning. In melt spinning, the properties of the fibers can be manipulated by adjusting various parameters such as extrusion (or spinning) rate, cooling rate, and control of the draw ratio at spinning (Gupta, 1997). If the polymer cannot be melt, solution spinning can be applied, which widens the further choice of material. It should be noted that there are also various natural fibers, such as silk, wool, and cotton. In this respect, using off-the-shelf fibers for 3D fiber constructs would be much more beneficial, especially in terms of material selection.

In this study, we constructed a dual-layer PEG hydrogel with HD-PEG hydrogel on the top and LD-PEG hydrogel at the bottom, and a 3D fiber construct was embedded in the LD-PEG hydrogel layer. The mechanical properties of each component and the final composite hydrogel were evaluated. Material for the 3D fiber construct was chosen based on the interfacial bonding with the hydrogel. Finally, we checked the stem cell differentiation within the final composite hydrogel.

## Materials and Methods

### Materials

Four-arm PEG (20 kDa) and 8-arm PEG (10 kDa) were purchased from Jemkem (Plano, TX, USA). Anhydrous dichloromethane (DCM) was purchased from JT Baker (Phillipsburg, NJ, USA), and ethyl ether was purchased from Fisher Scientific (Waltham, MA, USA). Norbornene-methyl-amine was purchased from TCI (Tokyo, Japan), and α-chymotrypsin was purchased from VMR Life Science (Randor, PA, USA). Matrix metalloproteinase sensitive peptide linker (MMPs peptide linker, H_2_N-KCGPQGIWGQCK-amide) was purchased from BeadTech (Gyoung-gi, Korea). For cell culture, high glucose Dulbecco's Modified Eagle Medium was purchased from Corning Life Science (Tewksbury, MA, USA), and low glucose Dulbecco's Modified Eagle Medium was purchased from HyClone (Anaheim, CA, USA). The fetal bovine serum and antibiotic-antimycotic were purchased from Gibco (Grand Island, NY, USA). For RT-qPCR, we used the NucleoSpin® RNA kit (Macherey-Nagel, Düren, Germany) and TRI reagent (Invitrogen, Carlsbad, CA, USA). PrimeScript RT reagent kit and SYBR Premix Ex Taq II kit were purchased in TaKaRa (Kusatsu, Japan). PCR primers were purchased from Bioneer (Daejon, Korea). All other chemicals were purchased from Sigma-Aldrich (St. Louis, MO, USA) and used without further purification.

### Fabrication of LD- and HD-PEG Hydrogels

PEG hydrogels were prepared by using thiol-norbornene photo-click chemistry. First, we prepared norbornene modified 4-arm (PEG4NB) and 8-arm PEG (PEG8NB). The detailed procedures are provided in the Supplementary Material.

#### LD-PEG Hydrogel

Precursor solutions of LD-PEG hydrogel with different PEG4NB concentrations were prepared. The amount of dithiol linker was matched by the stoichiometric balance between NB and thiol moiety molar concentration. Typically for 6 wt% of PEG4NB aqueous solution, 7 mM of dithiol linkers and 1 mM of lithium phenyl-2,4,6-trimethylbenzoylphosphinate (LAP) were added. To control the degradability of hydrogels, the ratio between two dithiol linker (1,4-dithiothreitol (DTT) and MMPs peptide linker) was varied with the molar ratio of 100:0, 75:25, and 50:50. For the rest of the tests, we fixed the molar ratio of DTT and MMPs to 50:50. The precursor solution was placed between two glass slides (Gap size: 1 mm) and irradiated with UV light (365 nm, 5 mW/cm^2^) for 2 min. Then, the hydrogels were swollen to an equilibrium state in PBS (pH 7.4) at 37°C.

#### HD-PEG Hydrogel

Precursor solution of HD-PEG hydrogels was prepared with different PEG8NB concentrations. The amount of DTT was matched by the stoichiometric balance between NB and thiol moiety molar concentration. The final HD-PEG hydrogel was prepared with 25 wt% of PEG8NB and 10 wt% of poly(ethylene glycol)-diacrylate (PEGDA, 700 Da) aqueous solution containing 100 mM of DTT and 5 mM of LAP. The hydrogel was prepared with the same procedure as described for LD-PEG hydrogel.

### Characterization of LD- and HD-PEG Hydrogels

The compressive modulus of LD- and HD-PEG hydrogels were measured using Universal Testing Machine (Lloyd Instruments, Ltd., UK). The swollen hydrogels were cut with an 8 mm biopsy punch and compressed with a rate of 1 mm/min. The compressive modulus was determined from the slope in between 0.04 and 0.1 strain. The shear storage modulus of the hydrogels was measured in a strain-sweep oscillation mode under a normal force of 0.2–0.3 N using a rheometer (MARS III, ThermoScinetific, USA). For the enzymatic degradation test of LD-PEG hydrogel, the hydrogels were incubated in PBS containing 1 mg/mL of α-chymotrypsin at 37°C. The medium was replaced every 24 h, and the change of shear storage modulus during the incubation was compared with the original shear storage modulus.

### Fiber Pull-Out Experiment

In order to select a proper fiber material, nylon monofilament (NY, DEKATEL, INC., USA), PGLA-monofilament (PGLA-mono, Samyang, Korea), and silk fiber (SF, Youngjin, Korea) were embedded in the LD-PEG hydrogel. In detail, a single fiber was fixed in-between two glass slides (Gap size: 2 mm). The precursor LD-PEG solution (only DTT linker) was dispensed in the middle of the fiber and irradiated with UV light (365 nm, 5 mW/cm^2^) for 30 min. The length of the fiber embedded in the hydrogel was 20 mm, and the free portion of fiber was at least 50 mm on each side of the hydrogel. The fiber embedded hydrogel was mounted on the bottom grip, and the free portion of fiber was fixed on the upper grip of the Universal Testing Machine. Care has been done to maintain the length of the free portion of the fiber in between the upper and bottom grip to be 40 mm. The fiber was pulled-out at a rate of 1 mm/min with a 10 N load cell, and the load during the pull-out of fiber from the hydrogel was measured. The fiber diameter of NY, PGLA-mono, and SF was 197, 183, and 193 μm, respectively, eliminating the fiber diameter effect. The image of the fiber inside the hydrogel before and after the pull-out test was taken using an optical microscope (Eclipse LV100, Nikon, Japan). The cross-sectional image of the fiber embedded hydrogel was taken with FE-SEM (SUPRA 55VP, Carl Zeiss, Germany). The samples were frozen and fractured within liquid nitrogen, followed by freeze-drying.

### Preparation of 3D SF Construct Embedded Dual Layer Hydrogel

**Figure 1:**
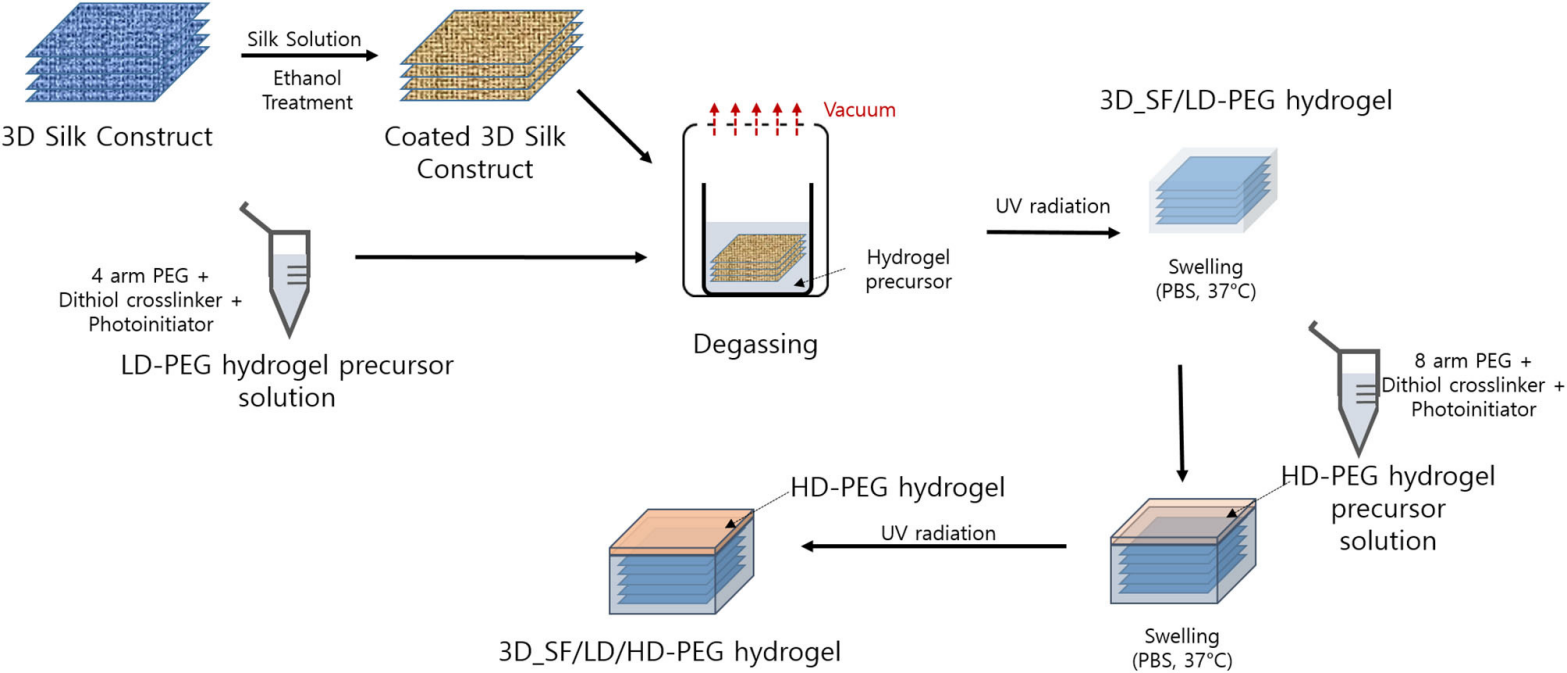
Overall process for the preparation of 3D silk fiber construct embedded dual layer hydrogel. LD-PEG, low-density PEG; 3D_SF/LD-PEG, 3D silk fiber construct embedded LD-PEG; HD-PEG, high density PEG; 3D_SF/LD/HD-PEG, 3D silk fiber construct embedded dual layer.

The overall preparation steps are summarized in Figure 1. Detailed procedures are described in the following sections.

#### Preparation of 3D SF Construct

The 3D SF construct was prepared using a pile frame. The pile frame was prepared by sticking the ballpoint steel pins in a pre-defined pattern on a PDMS mold (Supplementary Figure 3). By moving the SF around the piles, we created different patterns as shown in Supplementary Figure 4. Pattern I has been used throughout the following experiments. Once the 3D SF construct has been made, it was coated with a silk fibroin solution to give structural stability after removal from the pile frame. The silk fibroin solution was prepared according to the previous report (Kwak et al., 2017). In detail, the 3D SF construct, still on the pile frame, was wetted with silk fibroin solution using a paintbrush followed by degassing in a vacuum to enhance penetration of silk fibroin solution inside the SF. This procedure was repeated twice, but first with 3 wt% and second with 8 wt% silk fibroin solution. Further, the 3D SF construct was immersed in ethanol to insolubilize the silk fibroin coating layer. Finally, the 3D SF construct was dried and removed from the pile frame. The dimension of the 3D SF construct was 12 × 12 × 2 mm.

#### Preparation of 3D SF Construct Embedded LD-PEG (3D_SF/LD-PEG) Hydrogel

The 3D SF construct is now immersed in an LD-PEG precursor solution. Here, the LD-PEG precursor solution has an excess molar concentration of DTT to norbornene groups to generate 2 mM free thiol groups, allowing thiol-norbornene photocrosslinking with HD-PEG later on. The LD-PEG precursor solution with the 3D SF construct was placed in a vacuum in order to remove the air pocket in the 3D SF construct. Then, UV light irradiation (365 nm, 5 mW/cm^2^) was carried out for 2 min. Finally, the 3D_SF/LD-PEG hydrogel was swollen to an equilibrium state in PBS (pH 7.4) at 37°C and cut with a 4 mm biopsy punch. The final dimension of the 3D SF construct embedded hydrogel was ϕ4 × 2 mm.

#### Preparation of 3D SF Construct Embedded Dual Layer (3D_SF/LD/HD) Hydrogel

The 3D_SF/LD-PEG hydrogel was placed in between two glass slides, and the gap between the top of the hydrogel and the upper slide was adjusted to about 0.2 mm by adjusting spacer height. Then, the precursor solution of the HD-PEG hydrogel was injected into the gap, followed by irradiation of UV light (5 mM/cm^2^, 365 nm) for 2 min. After photo-crosslinking, the formed 3D_SF/LD/HD hydrogel was carefully detached from the glass slides. Finally, the dual-layer hydrogel was swollen to an equilibrium state in PBS (pH 7.4) at 37°C. The final dimension of 3D_SF/LD/HD-PEG hydrogel was ϕ4 × 2.25 mm.

### Characterization of 3D_SF/LD/HD Hydrogel

The compressive modulus of the 3D SF construct and 3D_SF/LD/HD hydrogel was measured using Universal Testing Machine. The compression rate of 1 mm/min was applied, and the compressive modulus was determined from the slope in between 0.04 and 0.1 strain. The time-dependent shear storage modulus change of the 3D_SF/LD/HD hydrogel was measured in a strain-sweep oscillation mode using a rheometer. Ten percent of strain was applied at a frequency of 60 times/min for 10 min.

### Human Mesenchymal Stem Cell (hMSC) Culture

#### Human Mesenchymal Stem Cell (hMSC) Culture

hMSC cells were cultured in a low glucose Dulbecco's Modified Eagle Medium containing 10% of fetal bovine serum, 1% of antibiotic-antimycotic, and 1 ng/mL of bFGF at 37°C and 5% CO_2_. For chondrogenic differentiation, hMSC were cultured with a differentiation medium of StemPro® Chondrogenesis Differentiation Kit for 14 days. All culture medium was replaced every 2–3 days.

#### hMSC Cell Encapsulation in 3D_SF/LD/HD-PEG Hydrogel

For 3D cell culture, we encapsulated hMSC in 3D_SF/LD-PEG hydrogel. Hydrogel precursor solutions were prepared with 6 wt% of PEG4NB, dithiol linkers (7 mM, molar ratio was 50:50 of DTT and MMPs) and 1 mM LAP. hMSC were suspended at a cell density of 2 × 10^6^ cells/mL in the hydrogel precursor solution, and 25 μL cell-suspended solution was carefully transferred into a cylindrical mold (diameter: 5 mm) containing the 3D_SF. The cell-suspended 3D_SF/LD-PEG hydrogel was prepared by exposing it to UV light (365 nm, 5 mW/cm^2^) for 2 min. Finally, HD-PEG hydrogel was deposited on the top of the cell-laden 3D_SF/LD-PEG hydrogel as described previously in section Preparation of 3D SF Construct Embedded Dual Layer (3D_SF/LD/HD) Hydrogel. The cell-laden 3D_SF/LD/HD-PEG hydrogel was cultured in the normal culture medium or differentiation medium for chondrogenesis. For 2D cell culture, hMSC cells were cultured on a cell culture dish (SPL, Korea) at a cell density of 5 × 10^3^ cells/cm^2^.

### Reverse Transcription-Quantification Polymerase Chain Reaction

Cell-laden hydrogels were collected and flash-frozen with liquid nitrogen for gene expression analyses. RNA extraction was performed using a NucleoSpin® RNA kit. Briefly, frozen gels were homogenized and incubated in 900 μL TRI reagent solution and mixed vigorously at room temperature for 5 min. Then the samples were filtered through NucleoSpin filters to clear lysates before adding 180 μL 1-bromo-3-chloropropane. Mixtures were vortexed for 30 s and centrifuged at 12,000 rpm for 10 min at 4°C for aqueous/organic phase separation. Subsequently, the colorless aqueous layers were transferred to clean microtubes, followed by the addition of equal volumes of 70% ethanol. RNA isolation and purification were conducted by following the manufacturer's protocol. Isolated RNA was converted into single-stranded cDNA using the PrimeScript RT reagent kit. Quantitative real-time polymerase chain reaction (PCR) was performed using the SYBR Premix Ex Taq II kit and a StepOne real-time PCR machine (Applied Biosystems, USA). The sequences of PCR primers for GAPDH, collagen II (COL2), Sox9, and aggrecan (AGG) are shown in the Supplementary Table 1. Samples were run at 95°C for 30 s, followed by 40 cycles of 95°C for 5 s, 55°C for 30 s, and 72°C for 30 s. Amplification of the SYBR signal was detected at the end of each cycle. Expression of target genes was normalized to that of GAPDH (internal control) using the 2^−ΔΔCT^ method.

### Statistical Analysis

All experiments were performed in triplicate, and the data are presented as the means ± standard deviations unless otherwise stated. For multiple-group comparisons, one-way ANOVA with the Tukey *post-hoc* test or Student's *t*-test was performed based on the results of the Shapiro-Wilk normality test and the *F*-test. According to the results, the statistical significances (^*^p < 0.05, ^**^p < 0.01, ^***^p < 0.001) of differences were determined between the indicated groups.

## Results

### Mechanical Properties of LD- and HD-PEG Hydrogel

**Figure 2:**
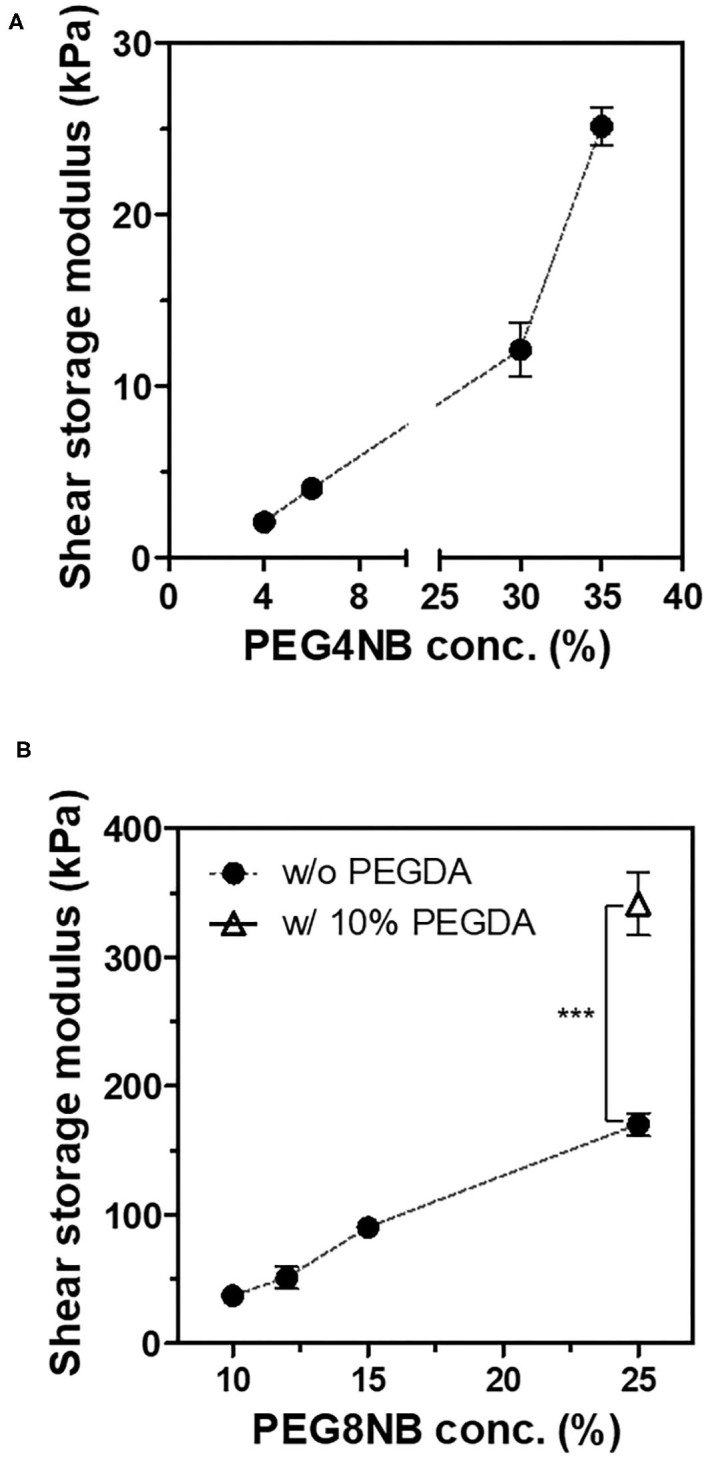
Shear storage moduli of PEG4NB **(A)** and PEG8NB **(B)** hydrogel according to its concentration and additives. ***p < 0.001 by Student's *t*-test.

First, we compared the shear storage modulus of PEG4NB and PEG8NB hydrogel (Figure 2). For PEG4NB hydrogel, the shear storage modulus increases with the concentration of PEG4NB because of the increased density of PEG chains. However, when the concentration of PEG4NB became higher, the viscosity of the precursor solution had increased also. The increase of viscosity would make the penetration of the precursor solution into the 3D fiber construct difficult, which could be problematic during the embedding of the 3D fiber construct into the precursor solution. Therefore, we fixed the PEG4NB concentration to 6 wt% in the case of LD-PEG hydrogel.

**Figure 3:**
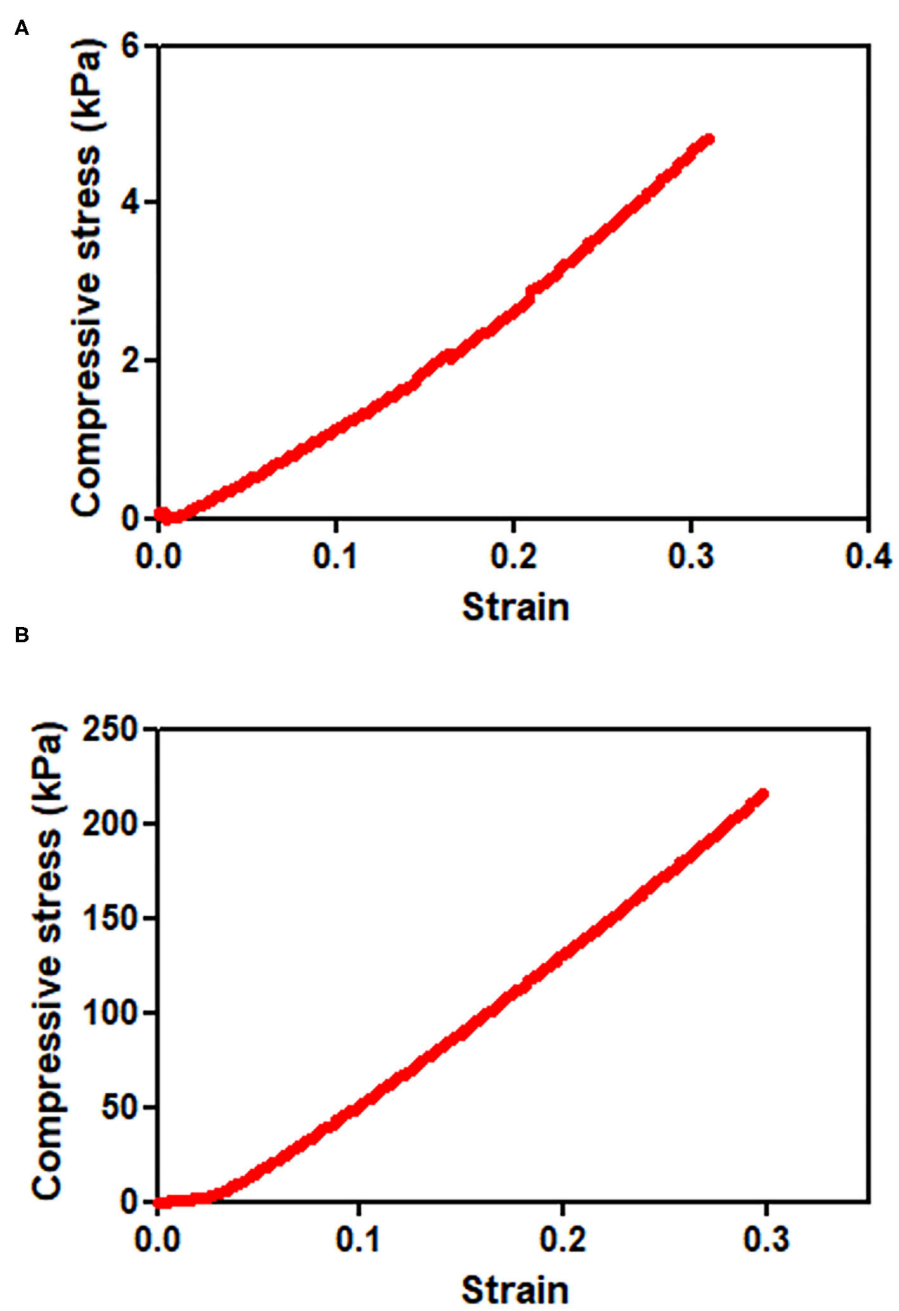
Compressive stress-strain curves of LD-PEG **(A)** and HD-PEG **(B)** hydrogel.

On the other hand, the PEG8NB hydrogel showed significantly higher shear storage modulus than the LD-PEG hydrogel. Since PEG8NB has more arms and a shorter chain length than the PEG4NB, more cross-linking density is expected. However, the shear storage modulus of the PEG8NB hydrogel is still far low for cartilage repair applications. Therefore, we added 10 wt% of PEGDA to 25 wt% of PEG8NB to further increase the cross-linking density. As a result, the shear storage modulus of the PEG8NB hydrogel could be increased significantly, and we used this combination as an HD-PEG hydrogel. Representative compressive stress-strain curves of LD-PEG and HD-PEG hydrogel are shown in Figure 3. The average compressive modulus of each hydrogel was 13.2 ± 3.1 and 700.1 ± 59.8 kPa, respectively. The result indicates that the HD-PEG hydrogel, which would serve as a top layer of the dual-layer hydrogel, can have a protective role against compressive stress.

### Biodegradability of LD-PEG Hydrogel

**Figure 4:**
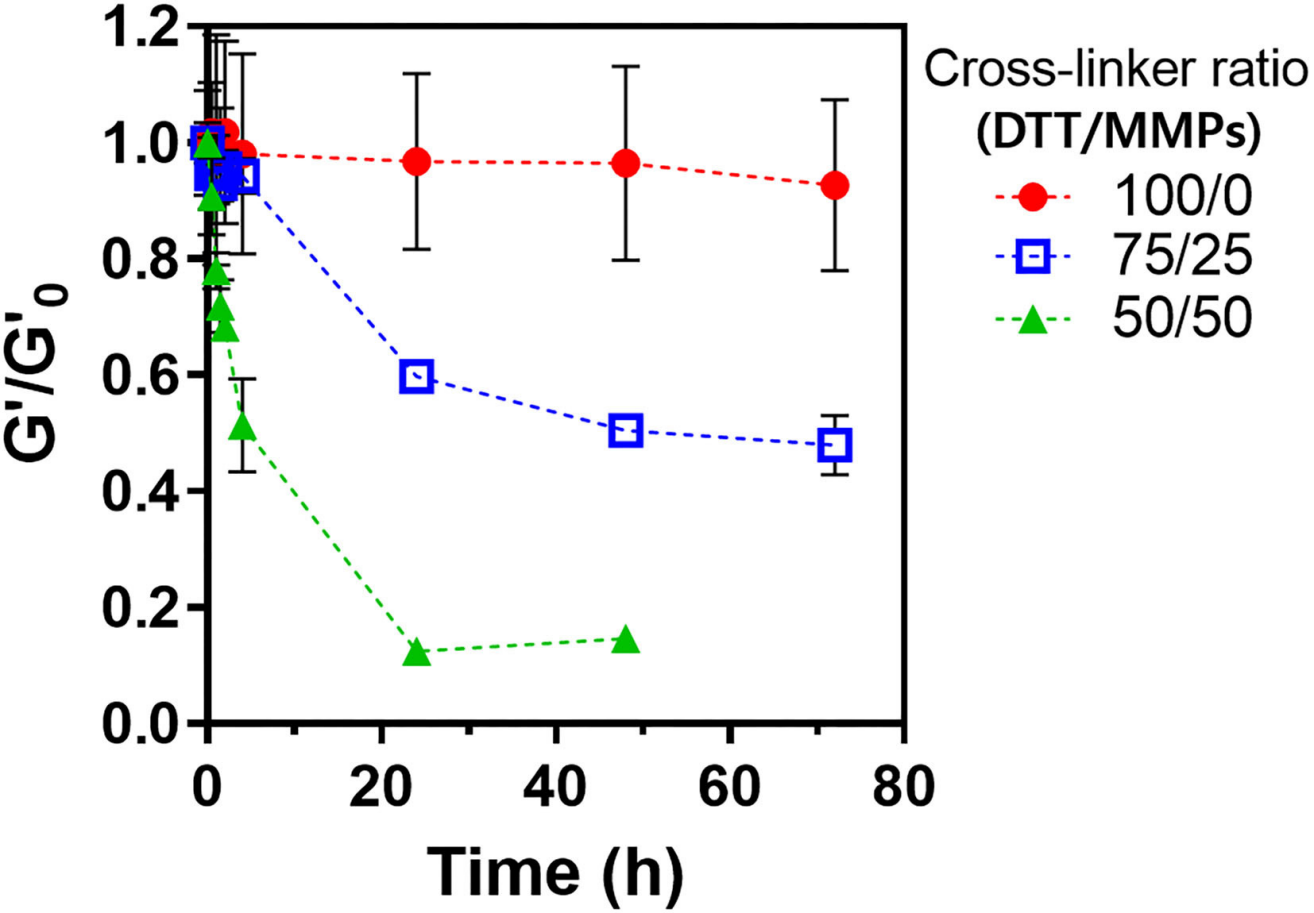
Shear storage modulus change during enzymatic degradation of LD-PEG hydrogel prepared with different dithiol-crosslinker ratio. α-Chymotrypsin of 1 mg/ml was used as a model enzyme. G'_o_: initial shear storage modulus of each hydrogel.

The LD-PEG hydrogel was designed to be the bottom layer of the dual-layer hydrogel. Therefore, it should have a controlled biodegradability to allow reconstruction of the cartilage tissue. We cross-linked PEG4NB with DTT and cysteine-containing peptide, which is non-degradable and degradable cross-linker, respectively. We used MMPs peptide linker for a degradable cross-linker where cysteine is incorporated on each N- and C-terminal, respectively. The degradation of different LD-PEG hydrogels was measured with the loss of the shear storage modulus of the hydrogel (Figure 4). As expected, when only DTT was used as a cross-linker, no degradation has occurred. When the MMPs peptide linker was incorporated, the degradation rate was dependent on the MMPs peptide linker content. We further tested whether the cell infiltration into LD-PEG hydrogel is possible. When the fibroblast was incubated on the LD-PEG hydrogel for 7 days, infiltration of the fibroblast into LD-PEG hydrogel was observed (Supplementary Figure 5).

### Fiber Material Selection – Pull-Out Test

**Figure 5:**
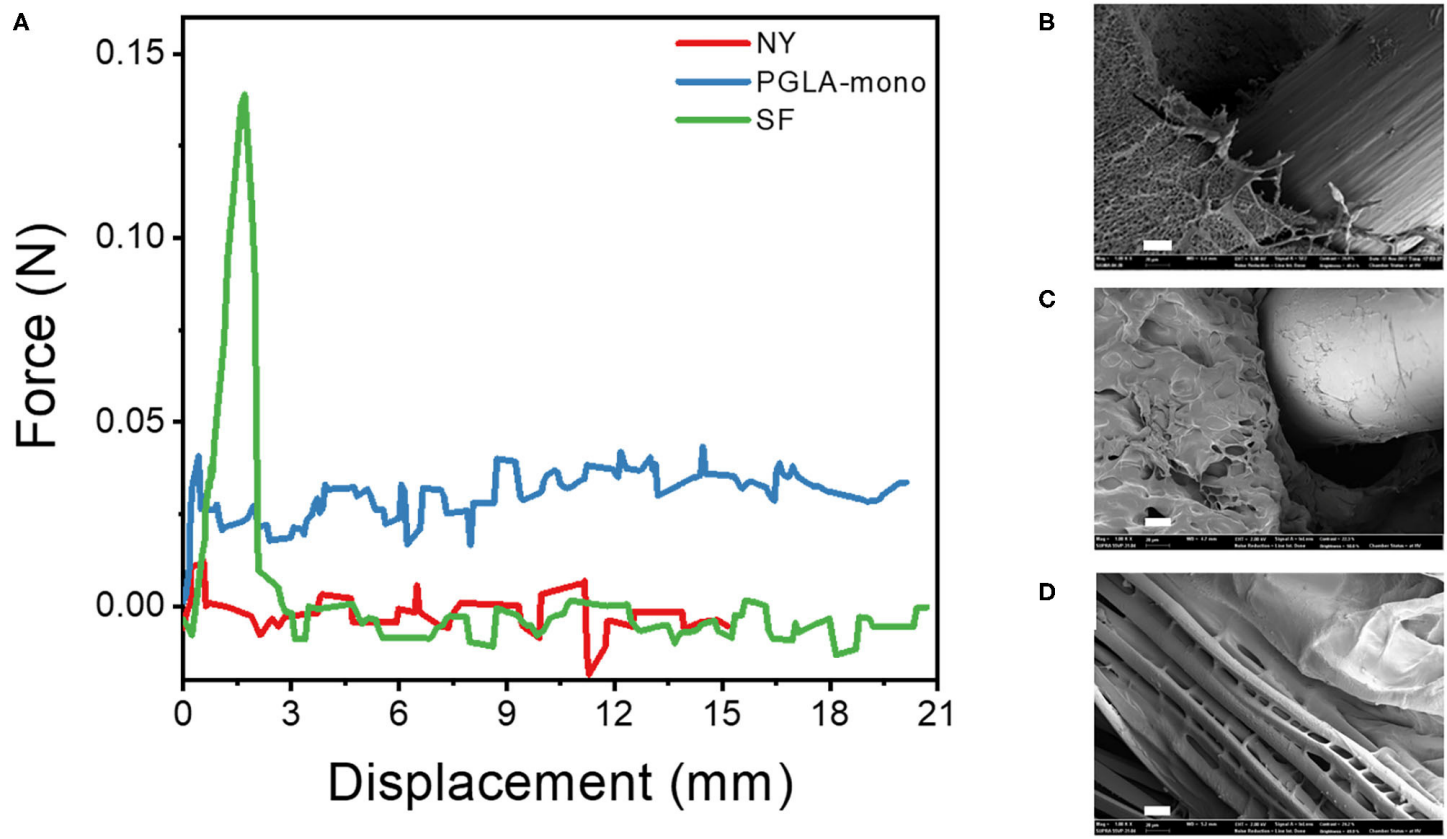
**(A)** Force-displacement curve during fiber pull-out test from LD-PEG hydrogel and **(B–D)** FE-SEM images of the cross-sectional of PGLA-mono **(B)**, NY **(C)**, and SF **(D)** embedded LD-PEG hydrogel. NY, nylon monofilament; PGLA-mono, PGLA monofilament; SF, silk fiber (Scale bar: 20 μm).

As confirmed previously with the mechanical properties test, the LD-PEG hydrogel needs to be reinforced to be used for cartilage repair. To find the more suitable fiber material, we measured the force during the pull-out experiment of three different fibers, NY, PGLA-mono, and SF, embedded in the LD-PEG hydrogel (Figure 5A). The initial increase of the pull-out force corresponds to the debonding force required for the fiber to be pulled out from the hydrogel. After the debonding of fiber from hydrogel, the pull-out force drops and maintains almost constant due to the frictional sliding. As shown in Figure 5A, SF exhibited the highest debonding force compared to NY and PGLA-mono, indicating a strong interfacial bonding force between SF and LD-PEG hydrogel. Even more, the LD-PEG hydrogel was damaged after the SF was pulled out, while nylon left the hydrogel clean and intact (Supplementary Figure 6). The cross-section of the fiber embedded hydrogel observed by FE-SEM also indicates strong adhesion of LD-PEG onto SF (Figures 5B–D). In PGLA-mono and NY, there was a clear separation between the hydrogel and the fiber, indicating weak or no interfacial bonding between them. However, when SF was embedded, adhesion of LD-PEG on the surface of silk fiber can be observed due to the strong interfacial bonding. Therefore, we have chosen the SF for materials to reinforce the LD-PEG hydrogel.

### Preparation and Compressive Modulus of 3D Fiber Construct

**Figure 6:**
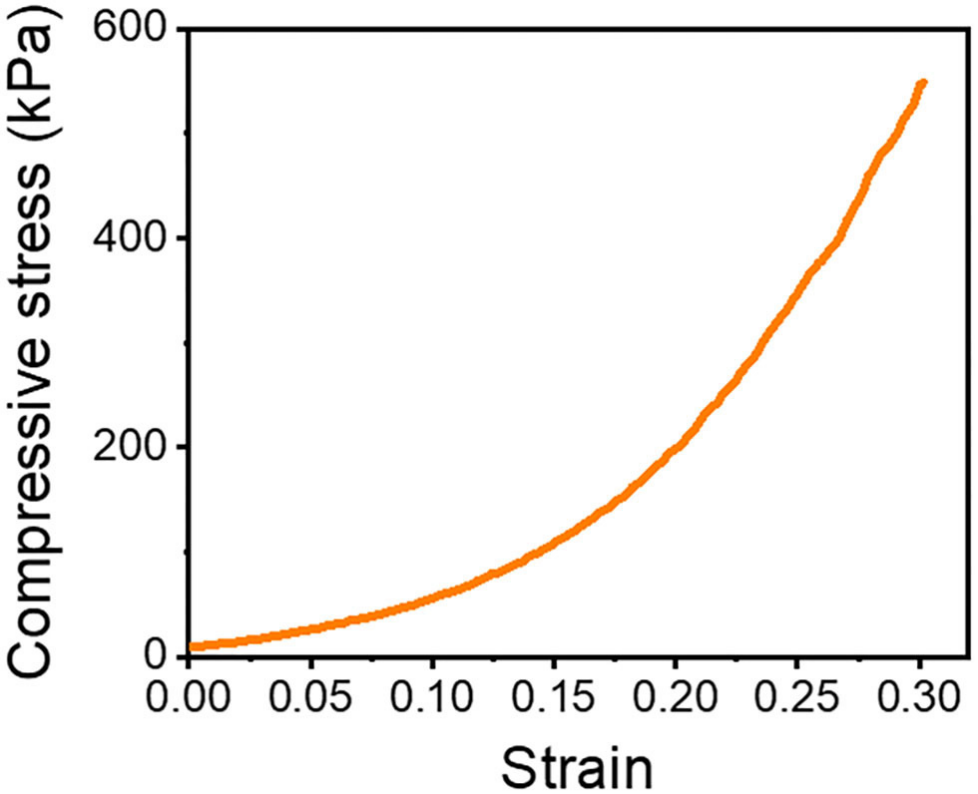
Compressive stress-strain curve of 3D SF construct having 208 crossing points. The 3D SF construct was coated with silk fibroin solution.

We prepared a pile frame to build the 3D SF construct, and by moving the silk fiber around the piles, we could develop different types of 3D SF constructs (Supplementary Figures 3, 4). A different set of pile designs, such as numbers and position of piles, allows the control of the number of silk fiber that overlaps. In this 3D fiber construct, the compressive modulus of the 3D SF construct relies on the number of overlapping points (Supplementary Figure 7). The representative compressive stress-strain curve of the 3D SF construct having 208 overlapping points is presented in Figure 6. The average compressive modulus of the 3D SF construct was 567 ± 94 kPa. It should be noted that the coating with silk fibroin solution was required to reduce slippage of SF at the overlapping point. Perfect prevention of the slippage could not be achieved, but handling stability during the experiments was greatly enhanced.

### Mechanical Properties of the 3D_SF/LD/HD-PEG Hydrogel

**Figure 7:**
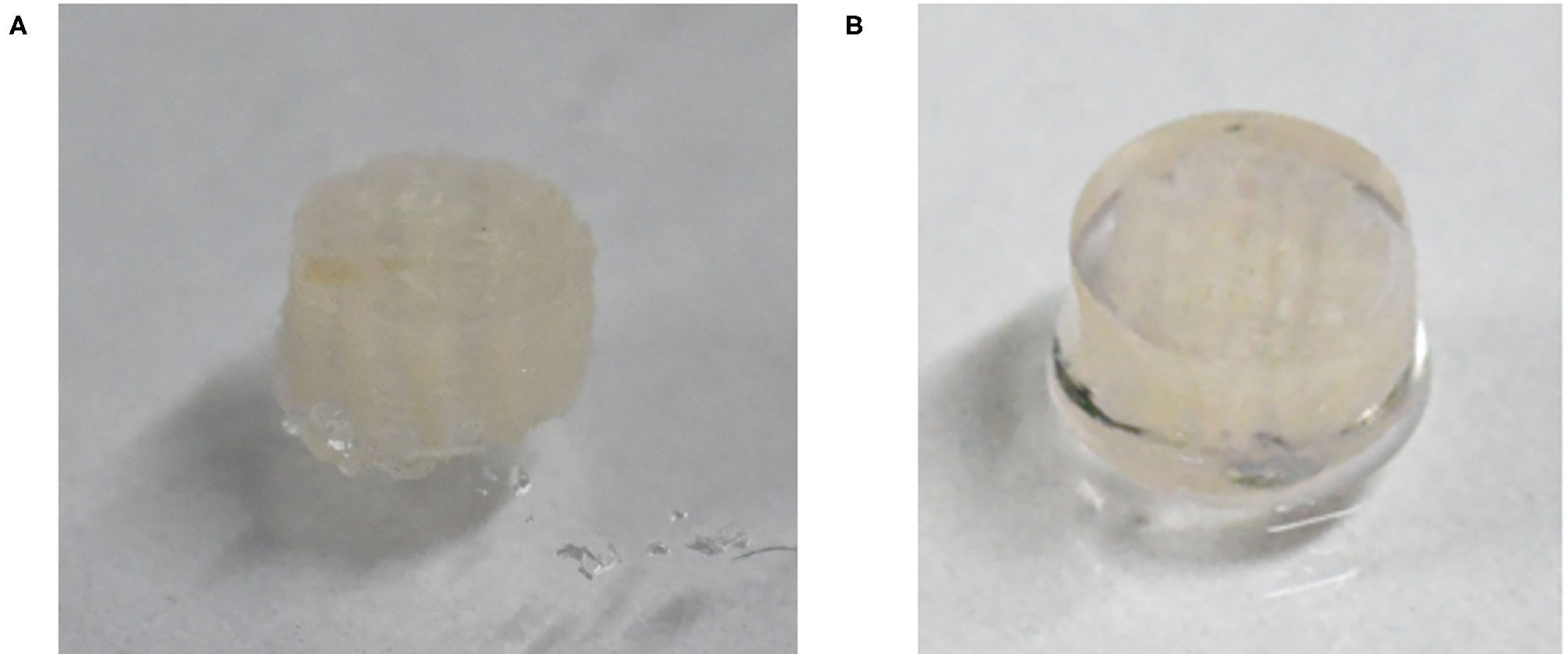
Optical image of 3D_SF/LD-PEG hydrogel **(A)** and 3D_SF/LD/HD-PEG hydrogel **(B)**. In the **(B)** image, for clear visibility of the HD-PEG hydrogel, the side of the 3D_SF/LD-PEG hydrogel was also covered by HD-PEG hydrogel.

**Figure 8:**
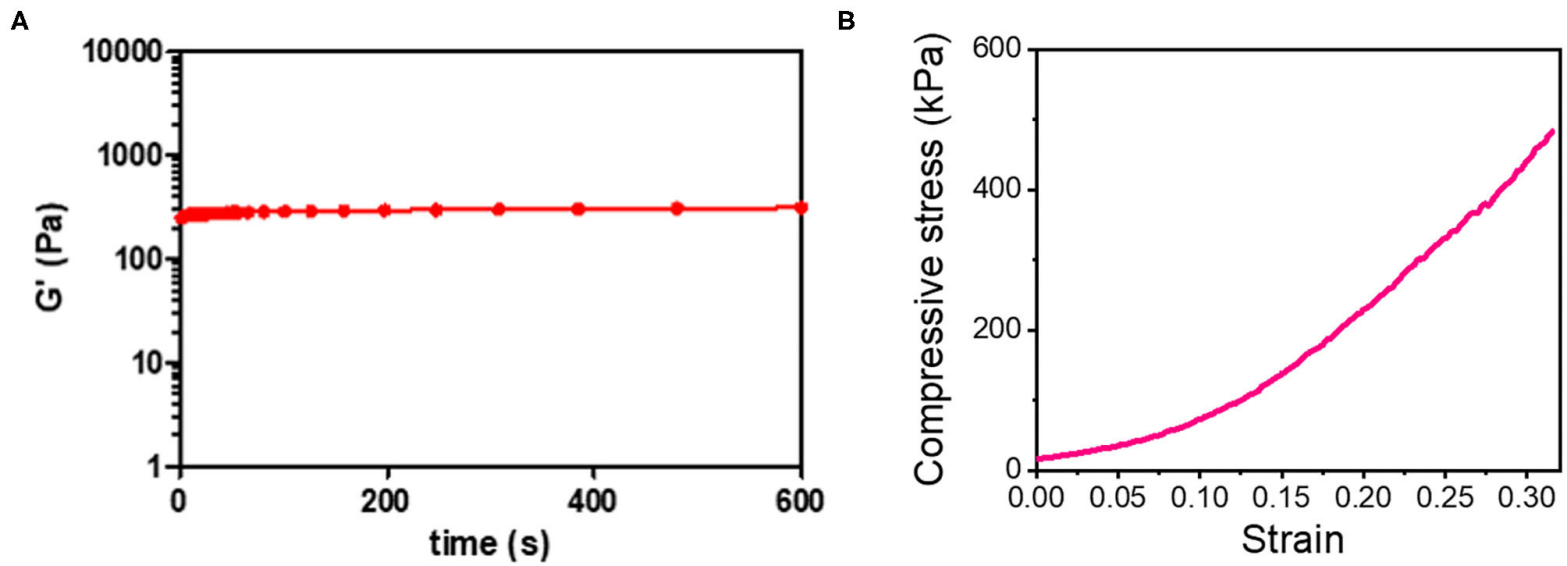
**(A)** Shear storage modulus of 3D-SF/LD/HD-PEG hydrogel when 10% of strain was applied in the frequency of 60 times/min for 10 min and **(B)** compressive stress-strain curve of 3D_SF/LD/HD-PEG hydrogel.

The optical image of the 3D_SF/LD-PEG hydrogel and 3D_SF/LD/HD-PEG hydrogel are presented in Figure 7. When combining different layers of a hydrogel, the interfacial bonding between the hydrogel layers is crucial. We left 2 mM of free thiol groups in the 3D_SF/LD-PEG hydrogel to photo-crosslink with the HD-PEG hydrogel. Figure 8A shows the shear storage modulus change of the 3D_SF/LD/HD-PEG hydrogel when 10% strain was applied repeatedly. The result shows no change of the shear storage modulus during the test, indicating stable interfacial bonding between all components; 3D SF construct, LD-PEG and HD-PEG hydrogel. The representative compressive stress-strain curve of 3D_SF/LD/HD-PEG hydrogel was presented in Figure 8B. The average compressive modulus of the 3D_SF/LD/HD-PEG hydrogel was 744 ± 133 kPa.

### Chondrogenic Differentiation of hMSC in the 3D_SF/LD-PEG Hydrogel

**Figure 9:**
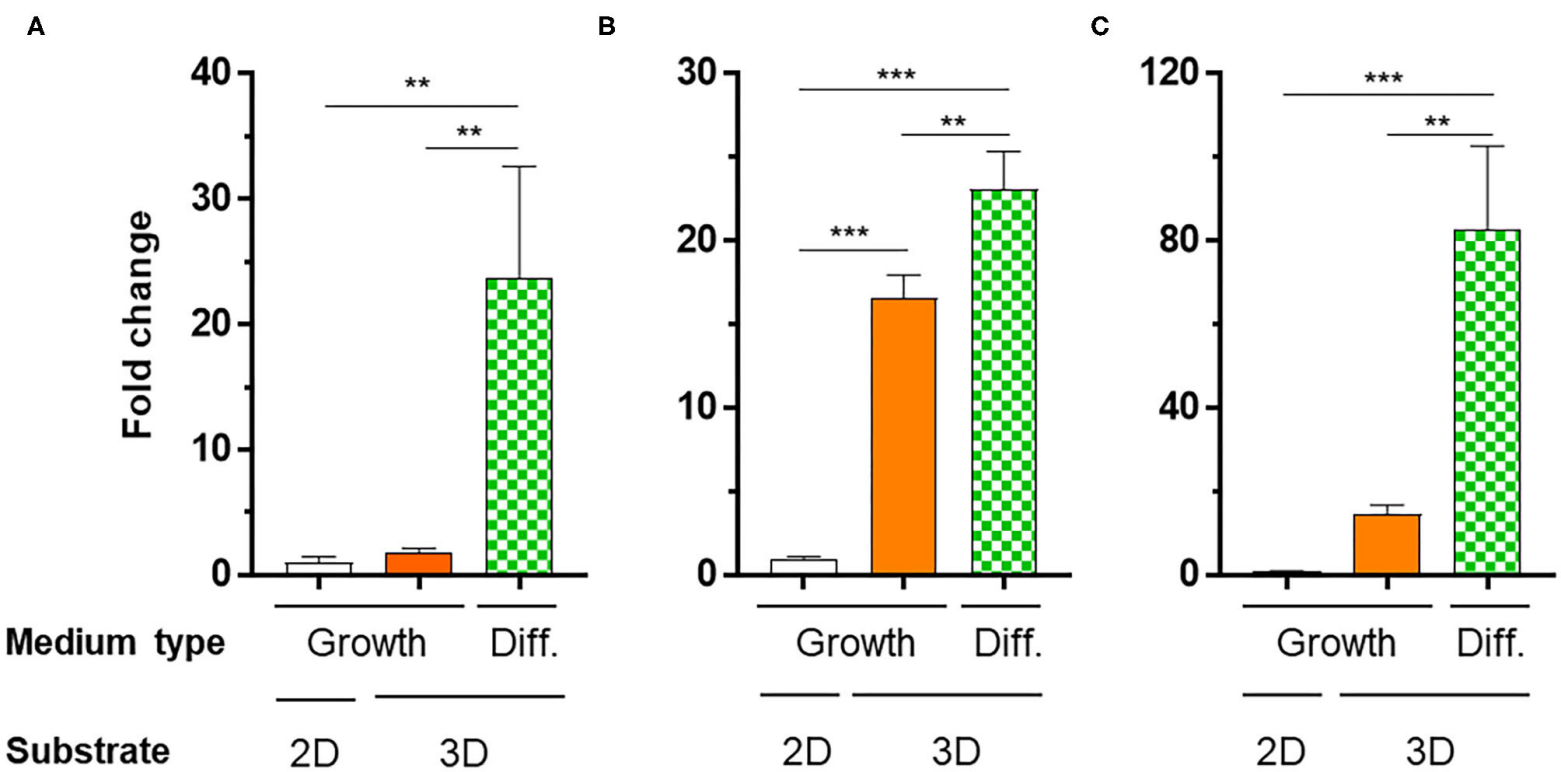
Relative mRNA expression levels of chondrogenic markers [**(A)** COL2, **(B)** SOX2, **(C)** AGG] of hMSCs cultured in different conditions. 2D, hMSC cultured on cell culture plate in normal medium (Growth); 3D, hMSC cultured in 3D_SF/LD/HD-PEG hydrogel either in Growth or differentiation medium (Diff.). One-fold indicates the expression level of a specific gene of cells cultured in Growth medium on cell culture plate. Statistical significance levels between indicated groups were obtained by one-way analysis of variance with Tukey multiple comparison test (**p < 0.01; ***p < 0.001).

To verify the potential of the 3D_SF/LD/HD-PEG hydrogel for chondrogenesis of stem cells, we encapsulated the hMSC in the 3D_SF/LD-PEG hydrogel. Live/dead assay after encapsulating hMSC in the LD-PEG hydrogel showed sufficient cell survival for further experiments (Supplementary Figure 8). Bright-field images of hMSC cells cultured in the 3D_SF/LD/HD-PEG hydrogel are shown in Supplemental Figure 9. The hMSCs were first cultured in standard growth medium for 7 days and additional 14 days in differentiation medium. After changing the growth medium to a differentiation medium, condensation of hMSC was observed. The chondrogenic differentiation of hMSCs was verified with chondrogenic markers (Figure 9). The expression level of chondrogenic markers of hMSCs cultured in standard medium on 2D cell culture plate was designated as 1-fold. When hMSCs were cultured in the 3D_SF/LD/HD-PEG hydrogel with standard medium, the expression of SOX9 and AGG have increased about 15-folds, indicating chondrogenic differentiation of hMSCs. Further, when the medium was changed to the differentiation medium, all chondrogenic markers, COL2, SOX9, and AGG, increased significantly. In particular, hMSCs in 3D_SF/LD/HD-PEG hydrogels under differentiation conditions showed an ~80-fold increase in AGG expression compared to hMSCs on 2D cell culture plates under normal growth conditions. These results indicate that the 3D_SF/LD/HD-PEG hydrogel provides a supportive environment for hMSCs to differentiate into chondrocytes.

## Discussion

It has been reported that the mechanical properties of PEG hydrogel are controllable by using PEG with different numbers of arms and concentrations (Lee et al., 2014; Kim et al., 2016). In this study, we adopted a thiol-norbornene PEG hydrogel which was previously used for studies on the effect of matrix elasticities and degradabilities on various cells (Lin et al., 2011; Wang et al., 2014; Sridhar et al., 2015; Kim et al., 2019). Therefore, we have chosen PEG4NB and PEG8NB as a candidate hydrogel precursor for LD- and HD-PEG hydrogel, respectively. The results of the shear storage modulus of LD- and HD-PEG hydrogel were in agreement with the above previous reports (Figure 2). However, PEG8NB alone did not have sufficient mechanical properties for HD-PEG hydrogel. Lee et al. (2014) have shown that the chain growth hydrogel (PEGDA) had better stiffness than the step growth hydrogel (PEG4NB or PEG8NB) due to the higher cross-linking density of the chain growth hydrogel. Therefore, we added PEGDA to improve the stiffness of HD-PEG hydrogel (Figure 2B). The mechanical properties of native articular cartilage have been reported previously. According to Spiller et al. (2011), the compressive moduli of mature cartilage are in the range of 300–800 kPa and the shear moduli of mature cartilage are in the range of 130–220 kPa. In another article by Little et al. (2011), the compressive moduli of native articular cartilage are in the range of 240–850 kPa under unconstrained compression mode and the shear storage moduli are in the range of 50–400 kPa. The compression and shear storage modulus of HD-PEG hydrogel were 700 (Figure 3B) and 330 kPa (Figure 2B), respectively, which are close to the upper limit of natural articular cartilage, showing that HD-PEG hydrogel can be used as a biomaterial for cartilage replacement.

The low stiffness of the LD-PEG hydrogel was reinforced by the 3D fiber construct. In fiber-reinforced hydrogel, the interfacial bonding between the hydrogel and the fiber is essential. A better interfacial bonding will result in better compatibility between the two materials. The strong interfacial bonding of silk fiber with the PEG hydrogel can be explained in several ways. First, silk is more hydrophilic than nylon and PGLA due to the polar and ionic side chains. It has been reported that blending silk fibroin with PGLA increase in hydrophilicity of the nanofiber mat compared to that of neat PGLA nanofiber mat (Zhou et al., 2015). Therefore, better interfacial compatibility is expected in the case of silk compared to nylon and PGLA. Besides, the penetration of PEG in between the filaments of SF has been observed (Figure 5D). It might be another reason for the high debonding force of SF from the LD-PEG hydrogel. The penetration of PEG in-between filaments increases the interfacial area, which further results in a positive effect on the cohesion of the fiber and the hydrogel.

Although we have chosen sutures as the candidate material for the 3D fiber construct, it was surprising that silk fiber exhibited the best compatibility with PEG hydrogel. Silk has been known as tough fiber due to its extensive β-crystallites network (Liu et al., 2016). In addition, silk is recognized as a promising biomaterial with low immunogenicity (Meinel et al., 2005). Due to the above features, there were many attempts to use silk as a biomaterial, and recently got FDA approval for some specific application (Melke et al., 2016). In articular cartilage repair, silk was considered as a candidate polymer due to its outstanding mechanical properties and cell affinity in the forms of electrospun fibers, hydrogel, and microfibers (Cheng et al., 2018; Fazal and Latief, 2018). However, the forms of silk proposed for these applications are prepared with the regenerated silk whose original structure of the silk has been destroyed. None of them showed the same mechanical properties as the native silk. Here, we used the native silk fiber, of which the original structural integrity was maintained. Another advantage of using native silk fiber would be the unique optical property of the silk fiber. Recently, Anderson light localization of silk fiber has been reported (Choi et al., 2018). Due to the parallel nanofibril arrangement, silk fiber can reflect incident sunlight. Such phenomena disappear when the orientation of nanofibrils is lost. Since our gelation acquires UV-light radiation of 365 nm, it is quite close to the visible light. Therefore, a reflection of the incident light by silk fiber would assist the photo-crosslinking efficiency of the PEG precursors. However, it should be verified by another study in the future.

In this study, we used a unique 3D fiber construct. The final outlook of the 3D fiber construct looks very similar to the FDM-based 3D fiber construct (Supplementary Figure 4). Previously, FDM 3D fiber constructs have been adopted as tissue-engineered cartilage constructs (Woodfield et al., 2005). In FDM, fibers are stacked as crossed “log-pile” construct by changing the direction of the nozzle head. Besides FDM, wet-writing (Agrawal et al., 2013; Puppi et al., 2016) and melt-electrospinning writing (MEW) (Visser et al., 2015) also resulted in a similar structure and applied as a hydrogel reinforcement. All these methods, the fiber spinning and construction of 3D layers are processed at the same time. However, limitations in materials choice and trial and error process would be the difficulties of these methods (Shahrubudin et al., 2020). In our case, we use off-the-shelf fibers and then construct the 3D layer. The advantage of our strategy is that we can use a wide range of fibers that already exist in the market, such as natural or synthetic fibers, mono- or multi-filaments, melt- or wet-spun fibers. Although we have shown only two different lay-down patterns (Supplementary Figure 4), various lay-down patterns can be created by using different set designs of piles and movement of fibers, which eventually affects the mechanical properties of the 3D fiber construct (Hutacher et al., 2001). The limitation of our methodology would be the fixation of the 3D fiber construct. We solved the problem by coating the 3D SF construct with a silk fibroin solution. Since the compressive modulus depends on the stacked crossing points of fibers, any fibers' slip at these points will not withstand the compressive stress. Indeed, the compressive modulus of the 3D SF construct with the coating (Figure 6) was higher than the non-coated one (Supplementary Figure 7B). Moreover, after embedding the 3D_SF in the LD-PEG hydrogel, further prevention of fiber slippage resulted in a higher compressive modulus of the final 3D_SF/LD/HD-PEG hydrogel (Figure 8B) than the coated 3D SF construct. These results suggest that our 3D fiber construct has its advantages when used to reinforce hydrogel.

Since we utilized three different components, strong interfacial bonding between each component is crucial. The strong interfacial bonding between the fiber and LD-PEG hydrogel has been shown previously. The remaining problem was how to bind the HD-PEG hydrogel on the top of the 3D_SF/LD-PEG hydrogel. Our solution was leaving some free thiol groups in the LD-PEG hydrogel that could participate during the photo-crosslinking of the HD-PEG precursor solution when deposited on the top of 3D_SF/LD-PEG hydrogel. A similar approach has shown effective in creating a strong interfacial adhesion of the bilayer hydrogel (Kinneberg et al., 2015; Aziz et al., 2017; Duan et al., 2017; Yang et al., 2020). As a result, the final 3D_SF/LD/HD-PEG hydrogel was stable even after multiple distortions (Figure 8A).

Another essential feature of the 3D_SF/LD/HD-PEG hydrogel is a microenvironmental niche for chondrogenesis of stem cells. The bottom 3D_SF/LD-PEG hydrogel has been designed to face the subchondral bone. Therefore, if microfracture is accompanied during the implantation, the LD-PEG hydrogel should allow penetration of bone marrow stem cells and cartilage reconstruction. As shown in Supplementary Figure 5, cell penetration into LD-PEG hydrogel was observed when the dithiol linker ratio was [DTT]:[MMPs] = 50:50. Further, the biodegradation rate was controllable by changing the dithiol linker's ratio (Figure 4). In general, implantable biomaterials should have an appropriate degradation rate depending on the implantation site (Williams, 2019). At the present stage, we will not say that the LD-PEG hydrogel with the selected ratio of dithiol linkers ([DTT]:[MMPs] = 50:50) has an adequate ratio of degradation for cartilage regeneration. However, since it is possible to control the degradation rate of the LD-PEG hydrogel, it has the potential to at least compensate for problems that may arise in future studies.

Another vital requirement for biomaterials is providing an appropriate elastic environment that favors mechanical signaling to the stem cell (Williams, 2019). Previous studies showed that the fate of stem cells is highly dependent on the stiffness of the hydrogel (Alakpa et al., 2016; Zhan, 2020). In a rigid hydrogel, stem cells express relatively high osteogenic markers, while in a soft hydrogel, neurogenic markers were dominated. In hydrogels with moderate stiffness of about 6 kPa, chondrogenic differentiation was observed previously (Zhan, 2020). The stiffness of LD-PEG hydrogel prepared from 6 wt% PEG4NB was chosen based on these previous studies. As shown in Figure 9, hMSCs cultured on LD-PEG hydrogel with 3D SF construct and HD-PEG hydrogel demonstrated successful chondrogenesis in that environment. The stem cell migration and differentiation behave differently in 2D and 3D culture systems. In general, a 3D culture system provides a better environment than a 2D culture (Vu et al., 2014; Bhattacharjee et al., 2015). Our results also indicate the effect of 3D culture when compared to the 2D culture (Figure 9). However, while the expression of Sox9 was positively related to the expression of aggrecan promotor (Figures 9B,C), it did not match with the expression of Col II (Figures 9A,B). Sox9 is a transcription factor that is essential for the expression of the type II collagen gene. It has been reported that the level of aggrecan promoter is highly related to the level of Sox9 (Sekiya et al., 2000). However, the level of Col II does not have such a relationship (Aigner et al., 2003). Similar results were reported in another article (Chen et al., 2017). Nonetheless, our 3D_SF/LD/HD-PEG hydrogel provided an appropriate chondrogenic microenvironment, which suggests the potential in articular cartilage repair.

In conclusion, we have successfully prepared a dual-layer composite hydrogel composed of LD- and HD-PEG hydrogel where the LD-PEG hydrogel was reinforced with the 3D SF construct. The composite hydrogel has sufficient mechanical properties required for articular cartilage repair. The bottom layer, composed of 3D SF construct and LD-PEG hydrogel, showed a controllable degradability and provided a positive chondrogenesis environment. It increases the potential of the composite hydrogel for the reconstruction of articular cartilage. Further *in vivo* assessments are in progress, and it will be reported in the near future.

## Data Availability Statement

The raw data supporting the conclusions of this article will be made available by the authors, without undue reservation.

## Author Contributions

JK performed the 3D fiber construct and all compressive modulus experiments. JC performed the hydrogel part and all storage modulus experiments. CK supervised the hydrogel part design and experiments and KL designed the concept, supervised the overall experiments, and wrote the manuscript. All authors contributed to the article and approved the submitted version.

## Conflict of Interest

The authors declare that the research was conducted in the absence of any commercial or financial relationships that could be construed as a potential conflict of interest.
