# Supplementary material for: 3D Silk Fiber Construct Embedded Dual-Layer PEG Hydrogel for Articular Cartilage Repair – In vitro Assessment
Source: Front Bioeng Biotechnol. 2021 Mar 24;9:653509. doi: 10.3389/fbioe.2021.653509 (PMC8024629; doi:10.3389/fbioe.2021.653509)
Supplement: Supplementary file 1 [file Data_Sheet_1.docx]

**Supplementary Materials**

3D Silk Fiber Construct Embedded Dual Layer PEG Hydrogel for Articular Cartilage Repair – *in vitro* Assessment

Jung Soo Kim^†,1^, Jaeho Choi^†,1^, Chang Seok Ki^1,2^, Ki Hoon Lee^1,2*^

^1^Department of Agriculture, Forestry and Bioresources, Seoul National University, Seoul, Republic of Korea

^2^Research Institute of Agriculture and Life Sciences, Seoul National University, Seoul, Republic of Korea

1. Synthesis of PEG4NB and PEG8NB

For photo-crosslinking, 4-arm PEG (20 kDa, Jenkem, USA) and 8-arm PEG (10 kDa, Jenkem, USA) were functionalized with norbornene groups. Briefly, 4-arm PEG (PEG4OH) or 8-arm PEG (PEG8OH) was dried for more than 3 days in a vacuum to remove moisture. The fully dried PEG (5 g) was dissolved in anhydrous dichloromethane (20 mL, DCM, JT Baker, USA) with 4-(dimethylamino)pyridine (DMAP) (0.5 eq. to hydroxyl groups of PEG) and trimethylamine (TEA) (3 eq.). The mixture was stirred at room temperature for 15 min to activate the hydroxyl groups of PEG under a nitrogen atmosphere. Then, p-nitrophenyl chloroformate (PNC, 5 eq.) was added dropwise into the PEG solution and the mixture was stirred for 24 h at room temperature under nitrogen atmosphere. Subsequently, PEG-PNC was precipitated in cold ethyl ether (Fisher Scientific, USA). Vacuum-dried PEG-PNC was dissolved in 20 mL DCM; norbornene-methyl-amine solution (5 eq., TCI, Japan) diluted in 5 mL of DCM was added to the PEG-PNC solution and incubated at room temperature for 6 h under a nitrogen atmosphere in the dark. Finally, PEG4NB or PEG8NB was collected by precipitation in cold ethyl ether and vacuum-dried. To remove impurities, vacuum-dried PEG4NB or PEG8NB was dissolved in deionized water and dialyzed against deionized water for 3 days using a cellulose acetate tube (molecular weight cut-off: 12-14 kDa for PEG4NB and 3.5 kDa for PEG8NB), followed by lyophilization. The introduction of NB on PEG was verified using ^1^H-NMR by the peaks around δ = 6ppm (Figure S1 & S2).


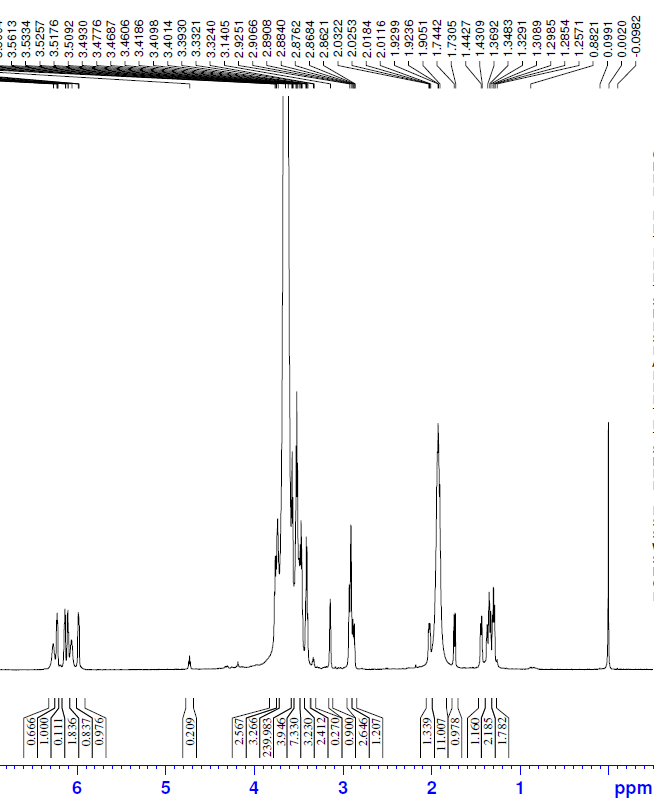


Figure S1. ^1^H-NMR of PEG4NB


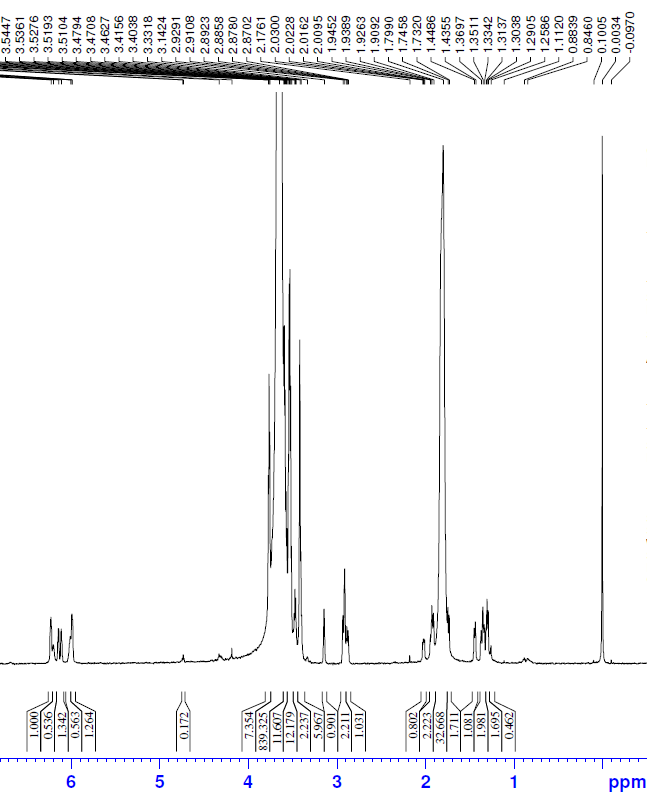


Figure S2. ^1^H-NMR of PEG8NB

2. 3D Fiber Construct

The 3D fiber construct was prepared using the following pile frames (Figure S3).


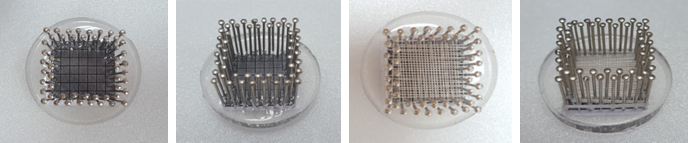


(A)


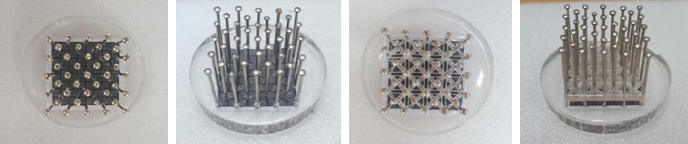


(B)

Figure S3. The pile frames for Pattern I (A) and Pattern II (B)

To prepare a 3D fiber construct, the fiber was moved around the piles as follows.


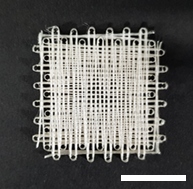


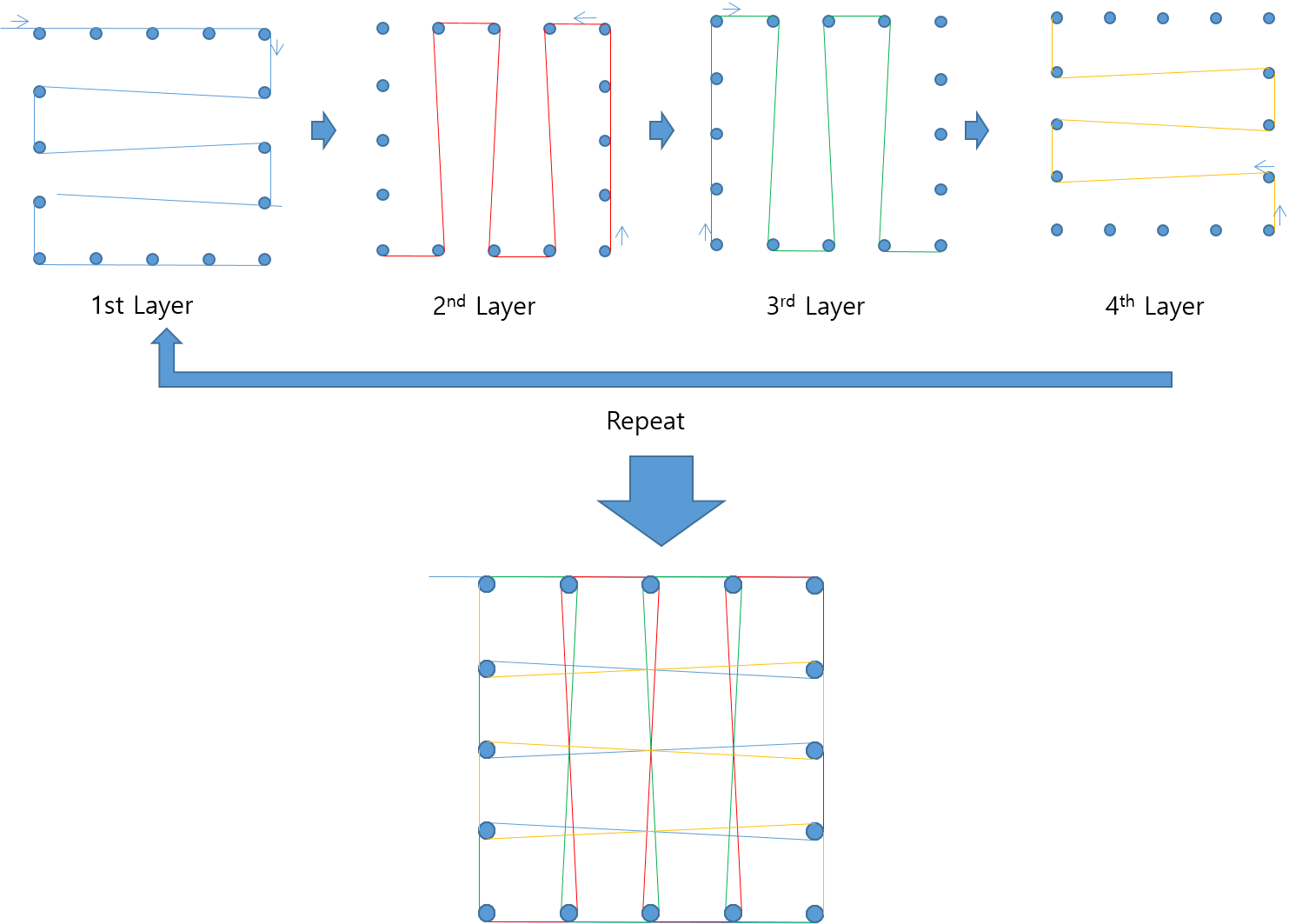


(A)


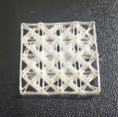


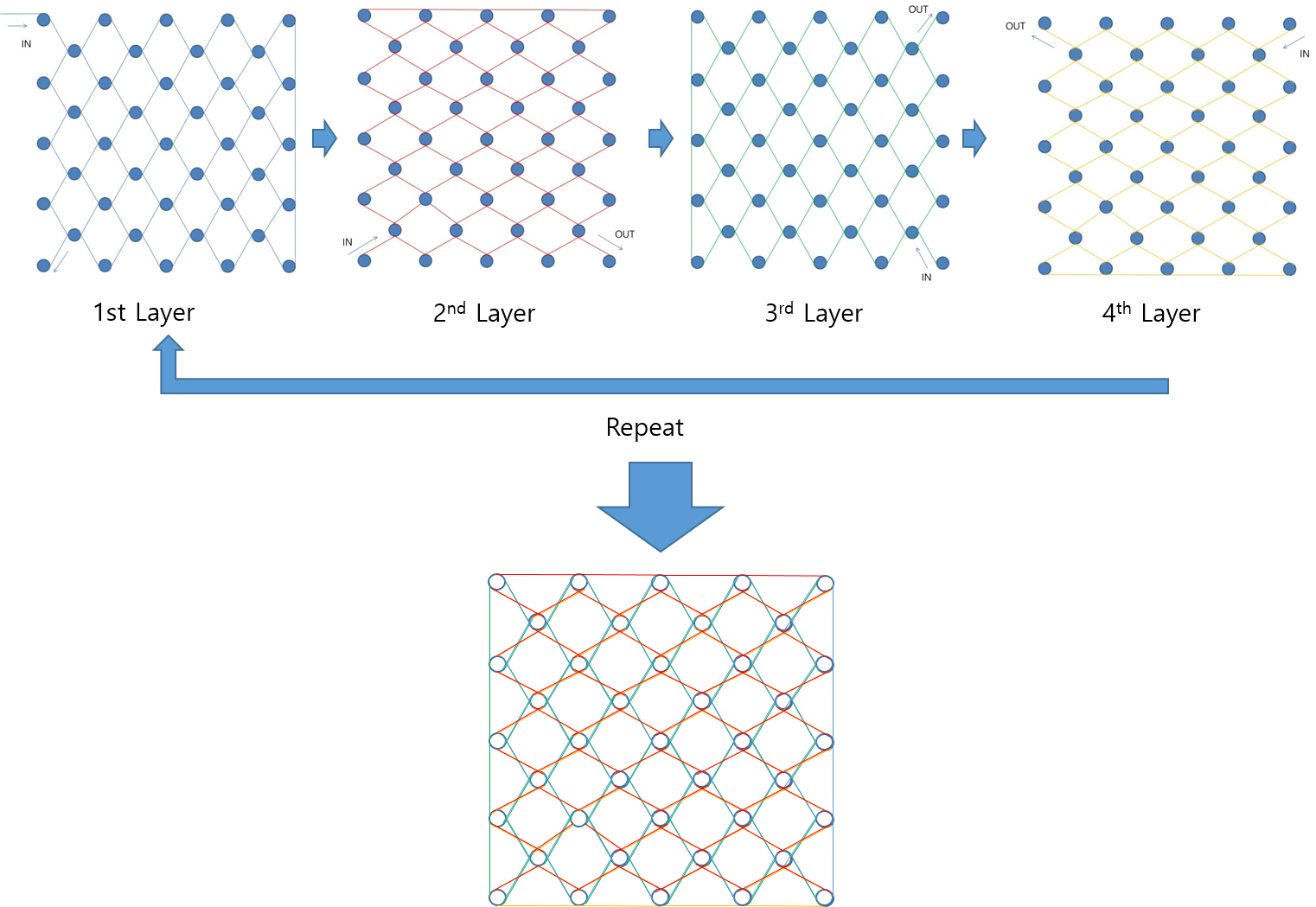


(B)

Figure S4. An example of the fiber movement around the piles; Pattern I (A) and Pattern II (B)

3. NIH-3T3 migration into PEG4NB hydrogels

Cell migration behavior into the PEG hydrogel was observed using a transwell system. 6 wt% PEG4NB hydrogels ([DTT]:[MMPs]=50:50) was placed on the upper chamber of transwell. NIH-3T3 cells were seeded on the hydrogel at a density of 3.3x10^5^ cells/cm^2^ and cultured for 7 days. NIH-3T3 cells were cultured in high glucose Dulbecco’s Modified Eagle Medium (Corning) containing 10% of fetal bovine serum (Gibco) and 1% of antibiotic-antimycotic (Gibco) at 37℃ and 5% CO_2_. While culturing cells, the gradient of the serum concentration inside and outside the transwell chamber was maintained. Subsequently, we obtained hydrogels and performed fluorescence staining. Briefly, the hydrogels were sunk in a paraformaldehyde solution (4 wt%) for immobilizing of cells and treated 0.1%(v/v) of triton X-100. Thereafter, washing hydrogels several times with PBS (pH 7.4), we stained NIH-3T3 cells with a phalloidin solution (4 U/mL) in which rhodamine was fixed and a DAPI solution (14.3 μmol/mL) for 1h. After washing the stained hydrogels with PBS (pH 7.4), the hydrogel cross-section was observed through a fluorescence microscope (Celena S, Logos Biosystem, Korea).


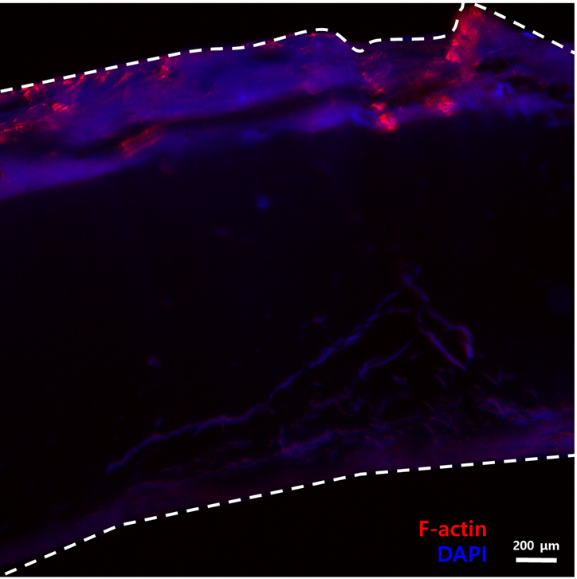


Figure S5. Cross-section of PEG4NB hydrogel after cell infiltration. F-actin can be observed on the top of the hydrogel.

4. Optical microscope image of fiber embedded LD-PEG hydrogel before and after fiber pull out test.


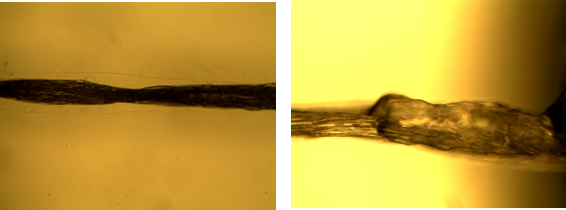


1. (B)


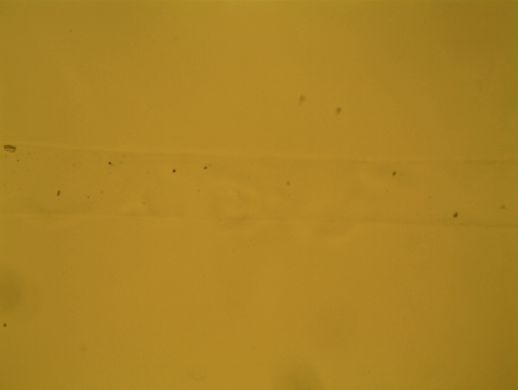


(C)

Figure S6. Optical microscope image of SF in LD-PEG hydrogel before (A) and after (B) pull-out test. NY embedded LD-PEG hydrogel after pull-out test (C)

5. Compressive stress-strain curves of different 3D SF constructs.

(A)

(B)

Figure S7. Representative compressive stress-strain curves of low-density (A) and high-density (B) crossing points in the 3D SF construct. The average compressive modulus of low-density and high-density overlapping 3D SF construct was 361 ± 57 kPa and 473 ± 128 kPa, respectively.

6. List of primer sequences used in this study.

Table S1. List of primer sequences used for RT-PCR analysis. F: Forward primer. R: reverse primer.

| Gene | Sequence |
| --- | --- |
| GAPDH | F : AATTCCATGGCACCGTCAAG  R : AGGGATCTCGCTCCTGGAAG |
| Collagen Ⅱ | F : GGCAATAGCAGGTTCACGTACA  R : CGATAACAGTCTTGCCCCACTT |
| Sox 9 | F : AGACAGCCCCCTATCGACTT  R : CGGCAGGTACTGGTCAAACT |
| Aggrecan | F : TCGAGGACAGCGAGGCC  R : TCGAGGGTGTAGCGTGTAGAGA |

7. Live/dead cell assay and optical images of cell in different growth medium.

For live/dead staining, stem cells were encapsulated and cultured within the LD-PEG hydrogel for one day. The cell-laden LD-PEG hydrogels were incubated in PBS containing 1 μM calcein AM and 4 μM ethidium homodimer-1 for 1 h and then washed with PBS. Cell morphology was observed using a fluorescent microscope (CELENA S; Logos Biosystems, Anyang, Korea), and fluorescence images were acquired by the z-stacking method (100-μm thick; 10 μm/slice) on at least four random areas of a single hydrogel.


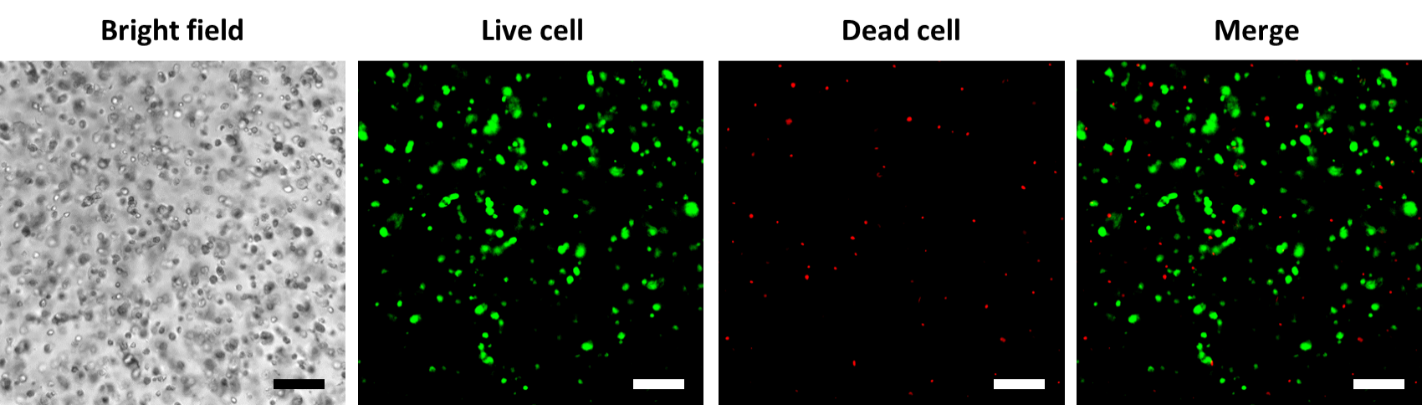


Figure S8. Live/dead cell assay on cell-laden hydrogel (scale bar: 200 μm)


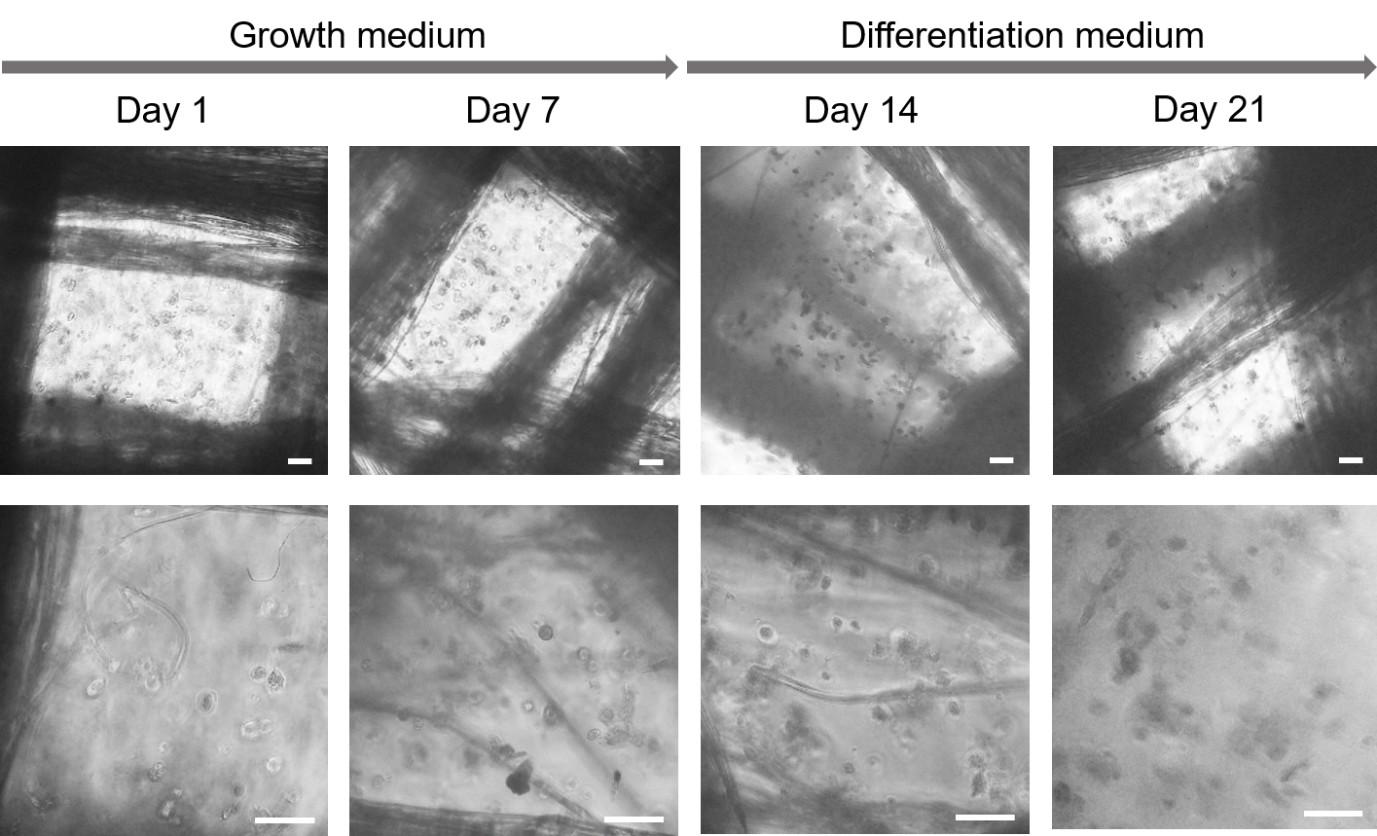


Figure S9. Optical microscope images of hMSCls cultured in the 3D_SF/LD/HD-PEG hydrogel. hMSC cell culture medium has been changed from normal growth medium to differentiation medium at day 7. Upper row presents low magnification images and bottom row presents high magnification images. (Scale bar: 10 μm)
